# Short Term Radiological Outcome of Combined Femoral and Ilium Osteotomy in Pelvic Reconstruction of the Child

**DOI:** 10.3390/children9030441

**Published:** 2022-03-21

**Authors:** Lorenz Pisecky, Gerhard Großbötzl, Stella Stevoska, Matthias Christoph Michael Klotz, Christina Haas, Tobias Gotterbarm, Matthias Luger, Manuel Gahleitner

**Affiliations:** 1 Department for Orthopedics and Traumatology, Kepler University Hospital GmbH, Johannes Kepler University Linz, Krankenhausstraße 9, 4020 Linz and Altenberger Strasse 96, 4040 Linz, Austria; gerhard.grossboetzl@kepleruniklinikum.at (G.G.); stella.stevoska@kepleruniklinikum.at (S.S.); christina.haas@kepleruniklinikum.at (C.H.); tobias.gotterbarm@kepleruniklinikum.at (T.G.); matthias.luger@kepleruniklinikum.at (M.L.); manuel.gahleitner@kepleruniklinikum.at (M.G.); 2 Department for Orthopaedics and Traumatology, Marienkrankenhaus Soest GmbH, Widumgasse 5, 59494 Soest, Germany; mcmklotz@gmx.net

**Keywords:** developmental dysplasia of the hip, neurogenic dislocation of the hip, Legg–Calvé–Perthes disease, combined osteotomy, femur, ilium, varisation derotation osteotomy, Salter, Pemberton, Chiari, open reduction

## Abstract

Background and Objectives: Reconstruction of the pelvic joint is a common way to address developmental dysplasia of the hip (DDH), as well as neurogenic dislocation of the hip (NDH) and Legg–Calvé–Perthes disease (LCPD) in children. The purpose of this study was to analyze the short-term radiologic outcome after hip reconstructive surgery either treated with sole osteotomy of the femur or in combination with iliac osteotomy in patients with DDH, NDH and LCPD. Materials and Methods: X-rays of 73 children, aged 2–18 years, with DDH, NDH and LCPD after hip reconstructive surgery were measured retrospectively and compared to the preoperative x-rays concerning various parameters to define hip geometry. The surgical procedures were femoral osteotomy (74), Salter innominate osteotomy (27), Pemberton osteotomy (27), open reduction (37), Chiari osteotomy (4). The pre-/postoperative acetabular index (AI), center-edge angle (CE) and Reimers migration index (RMI) were evaluated before and 3 months after surgery. Results: Hip geometry parameters improved significantly (RMI: preop/postop: 62.23% ± 31.63%/6.30% ± 11.51%, *p* < 0.001; CE: 11.53° ± 20.16°/30.58 ± 8.81°, *p* < 0.001; AI: 28.67° ± 9.2°/19.17 ± 7.65°, *p* < 0.001). Sub-group analysis showed a superior RMI in DDH compared with NDH 3 months after surgery (DDH/NDH: 2.77% ± 6.9%/12.94% ± 13.5%; *p* = 0.011). Osteotomy of the iliac bone (Salter innominate, Pemberton, Chiari) resulted in a significant improvement of the postoperative RMI compared to cases without osteotomy of the ilium (7.02 ± 11.1% vs. 16.85 ± 4.71%; *p* = 0.035). Conclusions: Femoral and pelvic osteotomies are effective to improve the radiological pelvic parameters in infants and adolescents with DDH, NDH and LCPD. In addition, the study found that the combination of femoral and pelvic osteotomy led to a better RMI than femoral osteotomy alone. Using the combined ilium and femoral osteotomy, it was possible to show the highest effect on correction of the hip geometry with respect to residual RMI.

## 1. Introduction

In infants and adolescents suffering from developmental dysplasia of the hip (DDH) and neurogenic dislocation of the hip (NDH), surgical treatment is often needed to avoid persistent problems in walking, standing and sitting [1]. Surgical hip joint reconstruction may be needed in children and adolescents with developmental dysplasia of the hip as soon as conservative treatment has failed and residual dysplasia is diagnosed to avoid persistent dysplastic morphology increase the risk of degenerative change [2].

In addition to DDH and NDH, in children with Perthes disease (LCPD) surgical reconstruction of the hip may be necessary to improve the containment. Especially if so-called ‘head at risk’ signs are present, literature postulates the concept of “super containment” [3,4,5].

Severe forms of DDH are often related to neuromuscular disorders [6]. In cerebral palsy (CP), different authors could show an incidence of NDH in up to 60% of their patients [7]. Therefore, hip reconstruction is even indicated in cases showing a progressive migration to avoid further dislocation and secondary complications of the hip [8,9,10].

Especially in adolescents and patients able to walk, therapy is often much more difficult than in smaller children because of shortening of extraarticular soft tissue [11]. Spasticity in neuromuscular disorders correlates with deformation and subluxation of the femur and shortening of the adductor muscle [12]. In most cases, a combination of procedures involving the bone and the soft tissue is needed to achieve complete reduction of the joint [13,14]. Common techniques are open reduction, lengthening of tendons or muscles as well as femoral and pelvic osteotomies [15].

Some surgeons prefer a two-stage approach to the reconstruction of the pelvic geometry and suggest performing surgical reduction and VDRO (varisation derotation osteotomy) first, in combination with an additional osteotomy of the ilium in a subsequent surgical procedure [16]. As long as there are not enough data to show superiority of the combined osseous procedure in patients with misalignment of the pelvic joint, the way of decision-making may still be unclear.

Available literature should have made clear, that a combination of femoral and iliac osteotomy is the proper choice in cases with femoral and iliac deformities. As the discussion is ongoing, whether to perform single or combined osteotomies as well as to perform those osteotomies stepwise and not within a single surgical procedure, the study group wanted to evaluate whether cases treated with combined one step osteotomy show different short term postoperative radiological outcome compared to cases treated with femoral osteotomy alone. The purpose of this study was to highlight the radiologic outcome after pelvic reconstruction either treated with osteotomy of the femur alone or in combination with iliac osteotomy in patients with DDH, NDH and LCPD.

## 2. Materials and Methods

In a retrospective study, patient files of children (aged 2–18 years) with DDH, NDH and LCPD were searched for hip or pelvic surgeries (open reduction, femoral/pelvic osteotomy ± soft tissue techniques) from 2008 to 2018 at a Central European University Hospital. Ethical approval was obtained from the IRB.

Patients without postoperative spica cast immobilization (3) were ruled out from analysis to reach homogeneity in aftertreatment. 10 patients were lost to follow-up. Finally, 73 patients (male/female: 38/35; 84 hips), aged 7.95 ± 5.18 years, were included. The group was treated by a single experienced pediatric orthopedic surgeon.

Indication for surgery can be seen in Table 1, demographic details and surgical procedures can be seen in Table 2. Included indications for surgery were RMI [17,18] 40 percent or higher or 25–40 percent with progression, classification of Tönnis [19] II or above or AI above the standard of Tönnis (greater than 25 degrees at the age of 2, >23° at 3, >20° at 7) [20]. Tönnis classification II or above for the AI-angle was an indication for surgery in respect of the lateralization of the ossific nucleus and a disruption of the Ménard–Shenton line. Surgery was not performed before the third year of age due to not having enough bone stock for extensive pelvic reconstruction.

The surgeries were carried out under general anesthesia on a radiolucent table using fluoroscopy. The patient was bedded in supine position with slight elevation of surgical site on by placing a foam padding beneath the iliac bone. The entire lower limb and the affected half of the pelvis were washed and draped. Surgical techniques used were open reduction, Chiari iliac osteotomy (4 hips) [21], Salter innominate osteotomy (21 hips) [22] and Pemberton acetabuloplasty (30 hips) [23], VDRO (84 hips) (Table 1) [24]. The surgical approach for open reduction and osteotomy of the ilium was direct anterior as described by Smith-Petersen [25]. A direct lateral approach was used to address the proximal femur for varisation-osteotomy. Beginning with open reduction and VDRO, additional osteotomy of the iliac bone was performed in cases with AI above the Tönnis-standard (Tönnis classification II or higher) and insufficient roofing of the femoral head after VDRO.

**Table 1:** Joints and surgical procedures [26].

	DDH	NDH	Perthes
N (hips)	31	25	28
age at surgery	5.8 years; 2.9–17; SD 5.7	12.5 years; 5.6–17.8; SD 6.2	7.6 years; 5.2–10.7; SD 1.7
m:f	5:21	11:11	22:3
right:left	10:11	12:7	9:13
bilateral	5	3	3
Surgical procedure in detail			
Femoral osteotomy	31	25	28
Osteotomy of ilium	27	17	12
Salter osteotomy	7	2	12
Chiari osteotomy	2	2	
Pemberton osteotomy	18	12	
Psoas tenotomy		9	1
Adductor tenotomy		8	
Open reduction	16	12	
Hamstring lengthening		7	
Lengthening of extension mechanism		1	

**Table 2:** Patient groups and surgical procedures in detail [27].

	Sole Osteotomy (Femur)	Combined Osteotomy (Ilium and Femur)
N (patients)	29	44
N (hips)	29	55
Bilateral	0	11
Gender m:f	9:20	28:16
Age at surgery	10.7 years; 5.0–17.8 y; SD 6.4	7.8 years; 2.9–15.1 years; SD 5.3
Location	10 right, 19 left	21 right, 12 left, 11 bilateral
Procedures (n = hips)		
Femoral osteotomy	29	55
Osteotomy of ilium	0	55
Salter osteotomy	0	21
Chiari osteotomy	0	4
Pemberton osteotomy	0	30
Psoas tenotomy	6	4
Adductor tenotomy	5	3
Open reduction	1	27
Hamstring lengthening	0	7
Lengthening of extension mechanism	0	1

The Salter innominate osteotomy was performed until the age of 8, as long as a good flexibility of the symphysis can be assumed. The procedure was not performed bilaterally in one surgical session.

The Pemberton acetabuloplasty was performed in cases with improper rounding of the acetabulum. Furthermore, the triradiate cartilage had to be clearly visible, which limited the procedure up to the age of 8. The procedure was performed bilaterally, if needed.

The Chiari osteotomy was considered to be a salvage procedure in cases with a lack of congruency of the pelvic joint (aspheric, big femoral head and small acetabulum) and progresses skeletal maturity. The procedure was not performed bilateral in one surgical session.

In a total of 55 cases, a combination of the techniques above was performed. Salter and Chiari osteotomies were fixed with three K-wires, Pemberton osteotomies were operated without osteosynthesis.

Osteosynthesis material used to hold the femoral osteotomy was a standard 90° AO blade plate (58 hips) or a 90° locking cannulated blade plate (15 hips).

Casting was performed directly postoperative under general anesthesia. Ten degrees of flexion, 10 degrees of inwards rotation and 20 to 30 degrees of abduction of the hip were tried to maintain within the cast. According to the routine protocol, immobilization was maintained for six consecutive weeks, followed by physiotherapy until full mobilization.

Complications occurring within the first three months after surgery were analyzed under usage of the full medical documentation and classified according to Clavien-Dindo [28]. Necessary unplanned procedures and admissions were collected and sub-group analysis concerning adverse events was carried out.

### 2.1. Radiological Analysis

The pre- and 3-months postoperative pelvic geometry data were analyzed twice each by author LP and compared statistically. An interval of at least 24 h between the two measurements was chosen. Intra-observer variation for the measurements was calculated using the mean difference, with the 95%-confidence intervals given. Intra-observer reliability rates were calculated using the statistical software Jamovi 1.0. Radiographic investigation consisted of a pelvis AP view in standing position and a lateral view pre- and 3 months postoperatively. AI [29], CE [29] and RMI [29,30] were measured.

### 2.2. Statistical Analysis

Statistical methods included detailed analysis of the epidemiological data with parameters for arithmetic mean, standard-deviation, median at continuous data and relative frequency for explained variables. The variables of interest for pre- and postoperative pelvic geometry were calculated using Student’s t-tests. Sub-group analysis was conducted for AI, CE and RMI pre- and 3 months after surgery. The statistical software Jamovi 1.0 was used.

## 3. Results

Following surgery, data for pelvic geometry improved in all analyzed groups (DDH, NDH, LCPD), and it was statistically significant (Table 3). The intra-observer reliability rates reached a mean of 0.953 (95%-CI 0.942–0.964).

In total, 28 hips showed a dislocation (RMI > 100%) before surgery. After open reduction and acetabular osteotomy, those cases presented a significant decrease of AI from 33.71° ± 7.76° to 19.71° ± 8.16° (*p* < 0.001).

**Figure 1:**
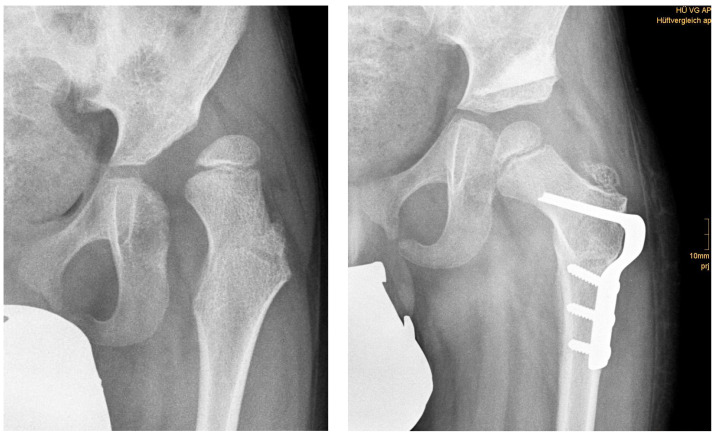
(**left**) Anteroposterior radiograph of a six-year-old male with NDH; (**right**) result 3 months postoperatively.

**Figure 2:**
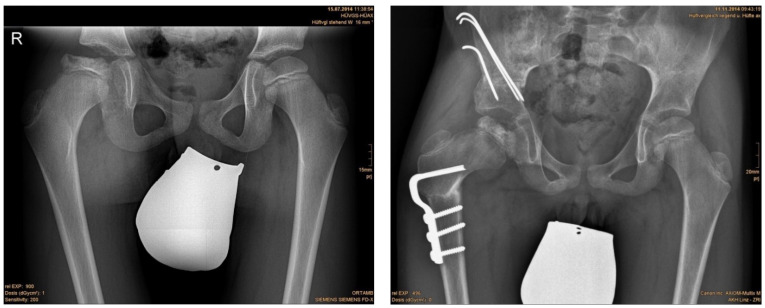
(**left**) Anteroposterior radiograph of a nine-year-old male with LCPD; (**right**): result three months postoperatively.

Three months after surgery, children with DDH showed significant decreased RMI compared with children with NDH in a two-sample Student’s *t*-test (2.77 ± 6.90% vs. 12.94 ± 13.50%; *p* = 0.011). More often an osteotomy of the iliac bone (Salter innominate, Pemberton, Chiari) was carried out in patients with DDH than with NDH (27 vs. 17; *p* < 0.001). In cases with combined one step iliac and femoral osteotomy, the residual RMI was significantly lower than in cases with osteotomy of the femur alone (7.02 ± 11.1% vs. 16.85 ± 4.71%; *p* < 0.001) (Table 4, Figure 1 and Figure 2).

In 30.01% of the patients (22), adverse events were found. Seven superficial skin lesions were counted, three extensive skin lesions, three spasticity of adductors, two subluxations, two dislocations of the cast, one deep infection of the osteosynthesis material, one compliance problem, one reluxation, one delayed bone healing and one spasticity of knee flexors. Classifying the adverse evenly according to Clavien-Dindo, 10 type I, 4 type II, 8 type III, 0 type IV and 0 type V events were seen. Short-term complications were observed nearly equally in the group of NDH (8/23; 34.7%) and in DDH (8/22; 36.4%). Further treatment of the complications included five procedures with three open re-repositions and re-casting as a result of re-dislocation within 12 weeks postoperatively observed on the follow-up x-rays after initial mobilization, one exchange of the osteosynthesis material due to a fracture of the blade plate, one removal of the plate due to deep wound infection involving the osteosynthesis material; three inpatient treatments for pain management and wound care and one admission for high energy shockwave treatment to address the prolonged healing of the bone were necessary. Four children needed intense wound treatment in the outpatient clinic on a regular basis. In no cases did very severe complications with persistent physical damage or even death occurred.

## 4. Discussion

Techniques in joint-preserving surgery in children and adolescents are demanding for the pediatric orthopedic surgeon. After walking age, treatment of DDH and NDH is difficult due to adaptive shortening of the extra-articular soft tissues, acetabular dysplasia, capsular constriction and increased femoral anteversion. In the literature several methods for reconstruction of the hip are available. In most of the children femoral and pelvic osteotomies are necessary to achieve concentric reduction. In cases of LCPD, the idea of ‘super-containment’ is well accepted to provide proper roofing for the rebuilding femoral head [3,4,5]. Realignment of the pelvic joint under usage of pericapsular osseous techniques in combination with or without femoral osteotomy and soft tissue procedures is a common way to treat DDH, NDH and LCPD in children.

The purpose of this study was to evaluate the short-term results of hip reconstructive surgery in young patients with DDH, NDH and LCPD either with ilium osteotomy alone or in combination with femoral osteotomy.

In this study-group the surgical results for reconstruction of the pelvic joint, using the Pemberton-acetabuloplasty, pericapsular osteotomy as described by Salter, Chiari osteotomy and varisation derotation osteotomy of the proximal femur are similar to the prior published data. In total, 84 hips were included in this trial; indications for surgical procedures were DDH, NDH and LCPD. For all three groups, it was possible to present a statistically significant enhancement in pelvic geometry following hip surgery. Therefore, our data suggest that these procedures are suitable to reestablish congruency of the hip in children and adolescents, despite the authors being aware of a progressive deterioration of RMI in cases of pelvic reconstruction at midterm follow-up [27].

The authors noticed that in this cohort, patients with NDH have a lower benefit from surgery in contrast to patients with DDH for the endpoint RMI (12.94% vs. 2.77%; *p* = 0.011). The results for RMI are congruent to previously reported data in children with NDH [14]. Further analysis revealed, that in cases with iliac osteotomy (Salter innominate, Pemberton, Chiari), the residual RMI was notable lower than in cases without (7.02% vs. 16.85%; *p* < 0.001). Iliac osteotomy was performed in 27 out of 31 patients with DDH, which is a notable contrast to the patients with NDH (17 out of 25). In the authors’ opinion, the osteotomy of the ilium leads to a significant improvement of RMI and should be performed to achieve congruency of the hip. As to our knowledge this is the first report to present advantages of the combined osteotomy for the endpoint RMI 3 months postoperatively. Clearly, a longer follow-up period is urgently needed to show the sustainability of this effect and will be published by the investigators.

Park et al. performed a similar retrospective trial, in order to find criteria for the one-stage femoral and ilium osteotomy [31]. As major risk factors for surgical failure, the authors revealed a preoperative RMI of 61.8% and above or 5.1% postoperatively. As the discussion is ongoing, in terms of what criterion to choose for concomitant osteotomy of the ilium, the RMI may be one possible option as well as the intraoperative stability of the reduced hip. In the authors’ opinion, the criteria of Park et al. involves a well thought-out approach to the discussion whether to perform a sole femoral osteotomy or a combined procedure.

Most notably, and supporting the ongoing dispute of surgical algorithm, Huh et al. pleaded for a stepwise approach to surgical management of decentration of the pelvic joint. At first, a combination of a proximal femoral osteotomy with open reduction and soft tissue techniques should be performed, followed by an osteotomy of the iliac bone in a second surgery, if needed [16]. In the authors’ opinion, one reason for thoughts such as this may be the good results of pelvic reconstruction under usage of sole osteotomies, whether femoral or iliac, as published by Czubak et al. 2018 [11], El-Sayed et al. 2015 [32] and Al-Ghamdi et al. did in 2012 [33]. It must be kept in mind, that especially in NDH, a deterioration of RMI is known and therefore the direct postoperative result in those cases should be as stable as possible and a combination of surgical techniques may be needed [14].

In this study, a rate of 30% for adverse events was calculated. Most of those events were easy to handle, which is on one hand comparable to previously published data, but way too high in the authors’ opinion [34]. It was expected to see more complications related to the postoperative immobilization such as contractures and superficial skin problems in the NDH group because of the higher muscle tone and the inability to express discomfort underneath the casting. Adverse events were more common in patients with NDH, despite the results not reaching statistical significance.

The heterogeneity of the investigated group as well as the small number of patients are the major limitations of the present study. The surgical techniques are not high-volume procedures, but necessary in a small patient group with demanding diseases.

The major drawback for statistical analysis, the heterogeneity, was accepted because the main result of the study, the improvement of RMI in combined femoral/iliac and sole femoral osteotomy, is not considered to be highly influenced by the underlying illness of the child at the 3-months postoperative follow-up. Clearly, the entity of the disease will make a difference in mid- to long-term follow-up trials. This must be considered when results for longer follow-up periods are given. Additionally, the authors are aware that the mid- to long-term complications may vary in those groups. Therefore, the authors cannot give recommendations for certain surgical procedures concerning long-term postoperative results. To minimize the chance for misinterpretation, all three sub-groups were analyzed separately concerning epidemiological data as well as the postoperative results to give the reader insights in preoperative clinical presentation and group-specific postoperative results. As prospective randomized clinical trials dealing with the described group of patients cannot be easily performed in the daily clinical practice, the authors must accept certain drawbacks in study design.

Furthermore, a limiting factor is the short postoperative follow-up period. The authors decided to evaluate the 3-month follow-up X-rays to achieve comparable short-term results for all evaluated patient groups (DDH, NDH, LCPD). Annual checks for deterioration of pelvic geometry are needed in NDH, as available reports show re-operation rates of 40% [35].

It is necessary to provide mid- and long-term results after hip reconstructive surgery to show long-lasting positive effects on hip geometry and patient outcome. The next step of the study group is to present mid- to long-term results on this topic and we hope to achieve sustainable results with our approach on hip deformities in children.

## 5. Conclusions

This study summarizes that all described reconstructive methods are feasible to enhance pelvic geometry in children with DDH, NDH and LCPD. In cases with iliac osteotomy, the residual RMI was significantly lower than in cases without.

The combined osteotomy of the ilium and femur led to a significant improvement of RMI compared to femoral osteotomy alone and should be undoubtedly performed in demanding cases. Common complications such as re-dislocation, wound-problems and delayed osseous healing were seen and led to demanding revision-surgery in five cases.

## Figures and Tables

**Table 3:** Pelvic geometry [27].

		DDH	NDH	LCPD	Overall
AI	pre	33.90 ± 7.30	29.61 ± 6.10	19.00 ± 5.30	28.67 ± 9.20
	post	19.43 ± 7.20	18.56 ± 8.60	16.71 ± 5.92	19.17 ± 7.65
	diff	14.47 (95%-CI 11.82–17.86)	11.05 (95%-CI 7.00–15.16)	2.29 (95%-CI 0.39–4.00)	9.5 (95%-CI 7.60–11.65)
	*p*-value	*p* < 0.001	*p* < 0.001	*p* = 0.019	*p* < 0.001
CE	pre	11.73 ± 10.40	14.30 ± 16.60	25.70 ± 6.10	11.53 ± 20.16
	post	31.70 ± 7.20	27.50 ± 12.0	32.90 ± 6.60	30.58 ± 8.81
	diff	19.97 (95%-CI 16.33–36.01)	13.2 (95%-CI 23.72–46.53)	7.2 (95%-CI 4.33–10.07)	19.05 (95%-CI 14.43–24.43)
	*p*-value	*p* < 0.001	*p* < 0.001	*p* < 0.001	*p* < 0.001
RMI	pre	80.28 ± 27.94	78.00 ± 21.4	27.38 ± 8.38	62.23 ± 31.63
	post	2.77 ± 6.90	12.94 ± 13.5	1.75 ± 4.14	6.30 ± 11.51
	diff	77.51 (95%-CI 65.8–89.43)	65.06 (95%-CI 48.68–73.08)	25.63 (95%-CI 22.36–29.24)	55.93 (95%-CI 48.01–62.85)
	*p*-value	*p* < 0.001	*p* < 0.001	*p* < 0.001	*p* < 0.001

AI—acetabular index, CE—center edge angle, RMI—Reimers migration index, DDH—developmental dysplasia of the hip, NDH—neurogenic dislocation of the hip, LCPD—Legg-Calvé-Perthes disease, pre—mean preoperative value, post—mean postoperative value, diff—mean difference.

**Table 4:** Improvement of RMI with and without osteotomy of the ilium.

		Sole Femoral Osteotomy (N = 29)	Combined Iliac and Femoral Osteotomy (N = 55)
RMI	pre	35.40 ± 14.60	72.00 ± 30.6
	post	16.85 ± 4.71	7.02 ± 11.10
	diff	18.55 (95%-CI 14.05–23.93)	64.98 (95%-CI 57.95–77.3)
	*p*-value	*p* < 0.001	*p* < 0.001
	diff	9.83 (95%-CI 6.59–15.32)	
	*p*-value	*p* < 0.001	

## Data Availability

The used datasets for this study contain potentially identifying or sensitive patient information. The vote of the local review board (Ethikkommission des Landes Oberösterreich, Wagner-Jauregg-Weg 15, 4020 Linz, Austria; Vote number EK Nr: 1183/2018) does not allow general data sharing.
